# Interaction between blood cadmium and lead concentration and physical activity on hypertension from the Korean national health and nutrition examination survey in 2008–2013

**DOI:** 10.1186/s12889-023-15614-x

**Published:** 2023-04-17

**Authors:** Jeoung A Kwon, Byungmi Kim, Eunji Kim, Kisung Kwon

**Affiliations:** 1 Institute of Health Services Research, Yonsei University, Seoul, Republic of Korea; 2 National Cancer Control Institute, National Cancer Center, Goyang, Republic of Korea; 3 National Cancer Center Graduate School of Cancer Science and Policy, Goyang, Republic of Korea; 4 Department of Environmental Medicine, College of Medicine, Ewha Womans University, Seoul, Republic of Korea; 5 Department of Sport Science, College of Art, Cultural & Engineering, Kangwon National University, Chuncheon, Republic of Korea

**Keywords:** Physical activity, Cadmium, Lead, Blood pressure, Hypertension, Interaction

## Abstract

**Background:**

Previous studies have suggested that blood Cd, Pb exposure, and physical activity levels may influence the development of hypertension. This study aimed to investigate the relationship between blood Cd, Pb levels, and hypertension by the level of physical activity in Korean adults using The Korea National Health and Nutrition Examination Survey (KNHANES).

**Methods:**

We used data from the KNHANES (2008–2013), a nationally representative, cross-sectional, population-based study. We included 8,510 participants who had records of blood Cd, Pb and, blood pressure measurements. Multiple logistic regression was used to examine the association between blood Cd and Pb exposure and the development of hypertension, as well as the modifying effects of physical activity levels. Additive interaction was estimated using relative excess risk due to interaction (RERI), attributable proportion due to interaction (AP) and synergy index (S).

**Results:**

Following covariates adjustments, we found significant associations of blood Cd and Pb with higher hypertension prevalence. This association was more apparent in low physical activity while blood Cd and Pb concentrations were not significantly associated with hypertension in participants with more activity. Additionally, there was a significant interaction between blood Cd and physical activity on hypertension risk (RERI = 0.17, 95% CI: -0.36–0.7; AP = 0.12, 95% CI: -0.28–0.52; S = 1.75, 95% CI:1.36–2.14).

**Conclusions:**

Our results suggest that low physical activity may substantially amplify the adverse effects of blood Pb and Cd exposure on hypertension risk. However, interactions were only found for Cd. Further studies are needed to confirm these findings.

**Supplementary Information:**

The online version contains supplementary material available at 10.1186/s12889-023-15614-x.

## Highlights

- This study is the first to investigate the interaction between heavy metals such as Cd and Pb and hypertension by physical activity level.
- Cd and Pb have an association with hypertension by accumulating in organs such as the kidneys and destroying vascular endothelial cells.
- It can be inferred that physical activity could reduce the amount of body Cd, Pb levels and reduce the negative effect on hypertension.

**Supplementary Information:**

The online version contains supplementary material available at 10.1186/s12889-023-15614-x.

## Introduction

Anthropogenic activities can produce heavy metals which can be environmental pollutants [1]. In addition, the general public may be exposed to naturally occurring heavy metals even if they are not occupationally exposed to heavy metals [2]. Heavy metals enter the body through means of air, water, and food and are absorbed [3]. Among heavy metals, Mn and Se are necessary in the human body for physiological and biochemical aspect [4], but Cd, Pb, and Hg are considered as nonessential metals which can cause health problems [4]. The negative effect of heavy metals on human health is increasing due to fast industrialization [3]. The amount of exposure of heavy metals is important in both essential and nonessential heavy metals [4]. Heavy metals that pass through blood, can accumulate in organs such as the kidneys, bones, liver, brain, and teeth [4–9].

Heavy metals such as Cd and Pb are toxic and therefore have a negative effect on the nervous, endocrine, renal, musculoskeletal, immunological, and cardiovascular systems, they are also carcinogens [7]. The effect of metal toxicity on the human body is Oxidative stress and DNA damage are negative outcomes of metal toxicity, metal toxicity can cause various diseases such as hypertension [10, 11]. Hypertension can be caused by both environmental and physiological factors, namely family history, alcohol consumption, smoking, age, physical activity, body mass index (BMI), food intaking, and stress [12]. Hypertension is one of the main risk factors of cardiovascular disease [13], Cd worsens hypertension by destroying vascular endothelial cells and reducing inflammatory mediators and antithrombotic substances [14, 15].

There are several epidemiological studies related to heavy metals and hypertension. Rodriguez-Iturbe et al. showed the positive relationship between heavy metal exposure and hypertension [13]. There were positive results in the association between blood Cd and increased blood pressure [13, 16, 17]. The amount of Hg exposure effects the relationship between blood Hg levels and hypertension. A positive relationship was identified between high level exposure of Hg and hypertension, however, there was no positive result in both low and moderate levels of exposure [18–21]. Pb and Cd are positively related to the risk of cardiovascular disease since they lead to problems with blood coagulation [22].

Physical activity is an important influencing factor in providing positive effects on the relationship between heavy metals and hypertension. The American Heart Association has identified physical activity as an important factor in coronary artery disease [23]. The most essential attributes in decreasing cardiovascular disease are exercise capacity and cardiorespiratory fitness (CRF) [24]. The American Heart Association CRF recommends CRF as it is positively related to risk management of cardiovascular disease [25]. CRF which measures the maximal oxygen consumption during physical activity means the ability of the circulatory and respiratory systems to provide oxygen to skeletal muscles while undertaking physical activity [25, 26]. No negative relationships between CRF and cardiovascular mortality were reported [25, 27, 28]. One of the important roles of PA is that it acts as a protective factor for various health problems, such as diabetes and metabolic diseases, as well as cardiovascular diseases such as chronic heart failure, coronary artery disease, arterial hypertension, hypercholesterolemia, and atherosclerosis [29–36]. Particularly in cardiovascular diseases, protection can be achieved by inducing partial or total changes in vascular metabolism, muscle, and fibrinolytic systems [35]. Also, in a study targeting metabolic syndrome, PA has a positive effect on endothelial cell dysfunction and oxidative stress [35]. PA increases the expression of nitric oxide synthase and EC-derived superoxide dismutase, and improves coronary EC function, vascular structure, and local and systemic fibrinolysis [37–39]. In addition, insulin sensitivity and EC function in insulin-resistant individuals can be improved by reducing body weight through PA [40]. It also reduces inflammatory cytokine levels through improvement of EC function [41], and PA make a positive impact on EC function by increasing the bioavailability of nitric oxide [42].

There are already many studies on the relationship between hypertension and physical activity, and heavy metals and hypertension [10, 11, 13, 23]. There are also studies that show that physical activity excretes heavy metals out of the body through sweat [43, 44]. Physical activity is one of the important health behavior. In terms of public health, physical activity plays a major role in health as the WHO emphasizes in “Global action plan on physical activity 2018–2030: more active people for a healthier world“ [45]. However, there are no studies on the effect of physical activity on the relationship between hypertension and heavy metals such as Cd and Pb. Therefore, we studied the relationship between blood heavy metals such as Cd, Pb levels, and hypertension by level of physical activity in Korean adults using The Korea National Health and Nutrition Examination Survey(KNHANES).

## Materials and methods

### Data source and study population

**Fig. 1:**
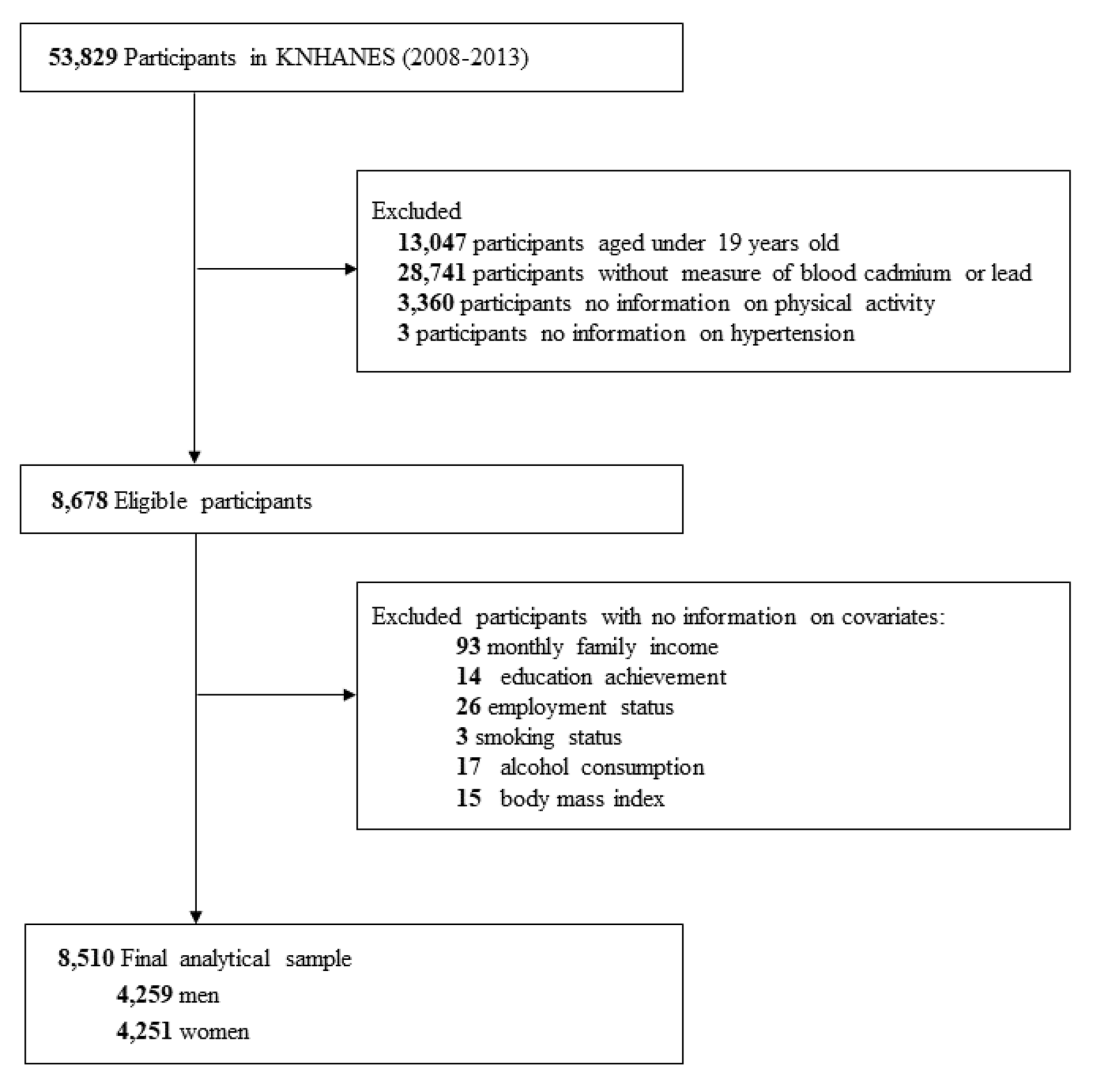
Flow diagram showing study sample

The study sample for this study was obtained from the KNHANES, which was conducted by the Korean Center for Disease Control and Prevention to assess the health and nutritional status of Koreans. The KNHANES is a cross-sectional survey of a nationally representative sample of Koreans that used a multi-step cluster probability design as the sampling strategy. More detailed protocols on the survey design, data collection and process for KNHANES has been published previously [46]. This survey collects a variety of information about demographic and socioeconomic factors, health-related behaviors, biochemical profiles, and clinical outcomes. One-third of the entire test group was randomly selected to provide blood lead and cadmium samples. Because heavy metal levels and blood pressure measurements were investigated only from 2008 to 2013, a total of 53,829 people who participated in this period were included in this study, 8,678 of whom met all of the following exclusion criteria: (1) those who aged under 19 years of age (n = 13,047); (2) those without measure of blood cadmium or lead (n = 28,741); (3) those with missing information on physical activity (n = 3,360) or hypertension (n = 3); (4) those with missing data (n = 168) on sociodemographic factors (educational achievement, monthly family income, employment status), and health behavior factors (smoking status, alcohol consumption, BMI). In total, 8,510 participants (4,259 men and 4,251 women) met the eligibility criteria for this study (Fig. 1).

### Measurement of physical activity

The validated International Physical Activity Questionnaire(IPAQ) was adopted to assess participants’ physical activity through the frequency, intensity, and duration of ‘Health enhancing physical activity’, ‘Minimally active’, and ‘Inactive’. There are two methods for calculating physical activity: categorical and continuous. Categorical scores have three levels of physical activity proposed to classify populations [47].

- ‘Health enhancing physical activity’ which is the most desirable level, one of the following two criteria need to be met [47]:

- Vigorous activity for at least 3 days, with > 1,500 MET-minutes per week, or.
- 3,000 MET-minutes per week for 7 or more days including activities such as walking, moderate activity, and strenuous activity combined.

(b) ‘Minimally active’ which is a case where one of the following three conditions is satisfied [47].

- 3 days or more of vigorous activity for 20 min or longer; or.
- >30 min of moderate activity or walking 5 days a week or.
- Any combination of walking, moderate activity, or strenuous activity with > 600 MET-min or more per week and 5 or more days per week.

(c) ‘Inactive’ which is the lowest level of physical activity, that does not meet the criteria of levels a or b [47].

The continuous score is a method of calculating and summing all activities. The unit is MET-min per week, and the calculation is ‘MET level of each activity’ × ‘minutes of each’ × ‘number of times per week’. 3.3 MET-min for walking, 4.0 MET-min for moderate-intensity activity, and 8.0 MET-min for vigorous activity [47].

### Cd and pb concentration measurements in whole blood

3 mL samples were placed in BD vacutainer tubes containing EDTA for trace element determination for measuring Pb and Cd (K2 EDTA tube, Vacutainers®; BectonDickinson, Franklin Lakes, NJ, USA). Graphite furnace atomic absorption spectrometry using Zeeman background correction was used for measuring Pb and Cd (Perkin Elmer AAnalyst 600; Perkin Elmer, Turku, Finland). The Neodin Medical Institute, a laboratory certified by the Korea Ministry of Health and Welfare conducted all blood metals analysis. Commercial references were used for internal quality assurance and control (Lyphochek® Whole Blood Metals Control; Bio-Rad, Hercules, CA, USA); the coefficients of variation of lead was 2.65–6.50% and cadmium was 0.95–4.82%. This institute passed the German External Quality Assessment Scheme (operated by Friedrich-Alexander University) and the Quality Assurance Program (operated by the Korea Occupational Safety and Health Agency) for external quality assurance and control. The Ministry of Labor which is one of the designated laboratories for analysis of specific chemicals such as heavy metals and certain organic chemicals certified the institute. The method detection limits of lead was 0.207 µg/dL and cadmium was 0.081 µg/L. All samples exceeded these detection limits.

### Blood pressure measurements and hypertension ascertainment

Blood pressure levels were measured in participants while rested after a 5-minute in a sitting position using the right arm with a mercury sphygmomanometer (Baumanometer Desk model 0320 in 2007–2012 and Baumanometer Wall Unit 33(0850) in 2013–2015, WABaum). For each participant, a blood pressure measurement taken on the right arm three measurements. Hypertension was defined as systolic blood pressure (SBP) ≥ 140 mm Hg, diastolic blood pressure (DBP) ≥ 90 mm Hg; self-reported doctor-diagnosed high blood pressure or the use of antihypertensive medications. Ascertained hypertension was used as dichotomous variable.

### Covariates

To examine the associations between exposure to blood Cd and Pb levels, physical activity, and hypertension, we included additional data such as demographic variables, lifestyle behaviors, and anthropometric measurements. Demographic factors including age, gender, education achievement, and monthly family income were assessed using a questionnaire. We classified the education level into two categories: less than high school, and college or more. Household income was classified into quartiles to adjust for the possible effect of income. Behaviors relevant to health, such as smoking status, alcohol consumption, and physical activity, were evaluated using a structured questionnaire and classified as categorical variables as follows: cigarette smoking status (Heavy smoker (≥ 5 packs), moderate smoker (< 5 packs), or never smoker); alcohol consumption (drinkers, or never drinker); and physical activity levels (inactive, moderate and most active). Anthropometric data including height and weight were also obtained, and the body mass index (BMI) was calculated by dividing the weight (kg) by the square of height (m^2^). Participants were stratified by BMI level into two groups according to the Asia-Pacific obesity classification for adult Asians as follows [48]: Acceptable weight (BMI < 23 kg/m^2^), overweight and obesity (BMI ≥ 23 kg/m^2^). Employment includes managers or professionals, office workers, service and sales workers, agriculture, fisheries or forestry workers, and blue-collar workers. The presence of diabetes was based on respondents’ self-reporting of a diagnosis by a doctor or the use of insulin treatment or defined as a fasting blood sugar level of greater than or equal to 126 mg/dL.

### Statistical analysis

KNHANES data were combined for all years from 2008 to 2013. All estimates in the present study were weighted analyses obtained through statistically weighted values provided by the KCDC, which was adjusted for the sample design and response rate [49]. The t test and chi-square test were used to compare characteristics between participants without hypertension and those with hypertension: age, educational achievement, monthly family income, employment status, smoking status, BMI, physical activity levels, alcohol consumption, blood concentrations of heavy metals (categorized into tertiles), presence of diabetes, and survey year. The age-adjusted geometric means and 95% confidence intervals (CIs) of blood Cd and Pb levels were calculated. Blood Cd and Pb levels were natural log transformed due to their right skewed distribution and categorized into tertiles (< first quartile, first to third quartile, ≥third quartile). Multiple logistic regression analysis for a complex sampling design performed using PROC SURVEYLOGISTIC to estimate ORs and 95% CIs for hypertension risk in terms of natural log-transformed blood Cd and Pb concentrations and tertiles of blood concentrations. We also performed stratified association analyses according to physical activity levels. Two models were discriminated. Model 1 was adjusted for age and sex. Model 2 was further adjusted for educational level, monthly family income, employment status, smoking status, alcohol consumption, BMI, physical activity levels, presence of diabetes, natural log-transformed different metals and survey year. Linear trends across tertiles were tested using multiple linear regression analysis. Moreover, additive interaction was estimated using relative excess risk due to interaction (RERI), attributable proportion due to interaction (AP) and synergy index (S) [50]. All statistical analyses were performed using SAS software, version 9.4 (SAS Institute, Cary, NC, USA), was used for data analysis. Statistical significance was considered as a value of *p* < 0.05.

### Sensitivity analysis

The logistic regression analysis has been widely used for complex sampling design. However, it could result in an overestimation of the effect of the exposure [51, 52]. To investigate the possibility of overestimation problem, we used PROC GENMOD and performed a binomial regression adjusting for the complex survey study’s weights, clusters, and strata.

## Results

Table 1 includes the baseline characteristics of the participants ≥ 19 years of age from KNHANES 2008–2013. The average age of the study participants was 43.59 ± 0.2 years, and 50.34% were male. Compared to inactive participants, participants with physical activity had higher BMI, alcohol consumption, were more likely to be male, employed, a heavy smoker, and of a high income level. The geometric means of blood Cd and Pb levels were 0.92 µg/L (95% CI, 0.91–0.94 µg/L) and 2.11 µg/dL (95% CI, 2.09–2.14 µg/dL), respectively, in all study participants. Moderately active participants tends to have lower levels of Cd and higher levels of Pb than inactive participants (Cd: 0.90 µg/L vs. 0.93 µg/L, Pb: 2.20 µg/dL vs. 2.04 µg/dL, all for p-value < 0.05).

**Table 1:** General characteristics of study participants

	N (%), Mean ± SE			
	Overall	Inactive	Moderate active	Most active
**Total**	8678	5342 (61.35%)	2674 (31.09%)	662 (7.56%)
**Blood Cd (µg/L)**				
Geometric Mean (95% CI)	0.92 (0.91, 0.94)	0.93 (0.91, 0.95)	0.90 (0.87, 0.92)	0.97 (0.91, 1.02)
**Blood Pb (µg/dL)**				
Geometric Mean (95% CI)	2.11 (2.09, 2.14)	2.04 (2.01, 2.07)	2.20 (2.16, 2.24)	2.38 (2.29, 2.48)
**Age**				
Age (year), Mean ± SE	43.59 ± 0.20	41.80 (0.29)	39.63 (0.33)	42.80 (0.72)
19–39	3624 (42.23)	2354 (43.65)	1134 (43.60)	222 (35.09)
40–59	3538 (41.17)	2041 (37.72)	1181 (44.26)	316 (49.36)
60+	1430 (16.60)	947 (18.63)	359 (12.14)	124 (15.56)
**Gender**				
Male	4359 (50.34)	2585 (43.90)	1814 (60.00)	482 (69.41)
**Educational achievement**				
College or more	3160 (34.51)	1981 (31.80)	996 (32.49)	183 (26.42)
**Monthly family income**				
High	2731 (30.48)	1763 (28.67)	1030 (33.13)	241 (34.01)
Middle	4741 (55.45)	3247 (56.33)	1636 (54.82)	382 (52.89)
Low	1113 (13.94)	799 (15.00)	341 (12.05)	89 (13.10)
**Employment status**				
Employed	5564 (64.40)	3191 (56.53)	1912 (69.25)	510 (75.18)
**Smoking status**				
Never smoker	4749 (54.85)	3169 (59.70)	1305 (48.70)	275 (40.87)
Moderate smoker (< 5 packs)	294 (3.29)	171 (3.11)	99 (3.52)	24 (3.75)
Heavy smoker (≥ 5 packs)	3632 (41.86)	2000 (37.19)	1270 (47.78)	362 (55.38)
**Alcohol consumption**				
Yes	6843 (78.31)	4179 (71.62)	2307 (76.40)	574 (82.20)
**Body mass index**				
Body mass index, Mean ± SE	23.75 ± 0.05	23.36 ± 0.06	23.76 ± 0.08	24.15 ± 0.14
Overweight/Obese (≥ 23 kg/m^2^)	3830 (43.80)	2986 (51.80)	1657 (55.32)	465 (63.31)
**Presence of hypertension**				
Yes	1972 (22.72)	1199 (21.49)	604 (20.34)	177 (24.47)
**Presence of diabetes**				
Yes	641 (7.30)	414 (7.08)	171 (5.69)	56 (7.76)
**Survey year**				
2008	1400 (15.74)	870 (14.72)	406 (13.49)	124 (15.92)
2009	1469 (16.79)	873 (15.16)	473 (15.30)	123 (17.67)
2010	1454 (16.84)	1027 (17.40)	523 (17.59)	130 (17.86)
2011	1469 (16.83)	1094 (18.49)	499 (16.43)	122 (16.82)
2012	1420 (16.15)	1027 (16.83)	515 (16.51)	112 (16.45)
2013	1466 (17.65)	990 (17.40)	613 (20.69)	107 (15.27)

Data are presented as weighted mean ± standard error or frequency (weighted percentage)

GM, geometric mean; CI, confidence interval; SE, standard error

Table S3 and Table S4 shows the age-adjusted blood Cd and Pb levels according to participants’ characteristics. For the overall population, blood Cd and Pb levels varied significantly differ according to age, gender, education achievement, smoking status, BMI, alcohol consumption, and survey year (p < 0.001). Blood Cd and Pb concentrations were significantly associated with age, gender, employment status, smoking status, and alcohol consumption in participants with hypertension.

The ORs and 95% CIs for hypertension for natural log-transformed blood Cd and Pb levels, tertiles of blood Cd and Pb concentrations, and physical activity levels are presented in Table 2. Compared with the lowest tertile, the ORs for hypertension were increased in the 2nd and 3rd tertiles of blood Cd and Pb in the unadjusted model. Following covariates adjustments, the ORs for hypertension remained elevated in the 2nd (Cd: adjusted OR = 1.84, 95% CI: 1.45–2.33; Pb: adjusted OR = 1.34, 95% CI: 1.08–1.66), and 3rd tertiles of blood Cd and Pb (Cd: adjusted OR = 2.46, 95% CI: 1.91–3.16; Pb: adjusted OR = 1.91, 95% CI: 1.48–2.45) (Nagelkerke R^2^ = 0.263).

**Table 2:** Association between heavy metal (blood Cd and Pb) concentration and risk of hypertension

		Hypertension		
Heavy metal		Model1		Model2
		Cases (%)	OR (95% CI)	OR (95% CI)
**Cd**				
Continuous			2.16 (1.93, 2.42)	1.77 (1.55, 2.02)
Q1 (< 0.64)		180 (10.90)	Reference	Reference
Q2 (0.64–1.39)		1077 (23.20)	2.26 (1.84, 2.79)	1.84 (1.45, 2.33)
Q3 (≥ 1.39)		715 (31.86)	3.43 (2.76, 4.26)	2.46 (1.91, 3.16)
*p*-value for trend			< 0.001	< 0.001
**Pb**				
Continuous			2.49 (2.10, 2.96)	1.7 (1.39, 2.07)
Q1 (< 1.60)		212 (13.74)	Reference	Reference
Q2 (1.60–2.81)		979 (21.51)	1.60 (1.31, 1.96)	1.34 (1.08, 1.66)
Q3 (≥ 2.81)		781 (33.85)	2.80 (2.24, 3.50)	1.91 (1.48, 2.45)
*p*-value for trend			< 0.001	< 0.001
**Physical activity levels**				
Most active		662 (26.10)	Reference	Reference
Moderate active		600 (22.42)	0.86 (0.67, 1.12)	0.99 (0.76, 1.29)
Inactive		1195 (23.27)	0.86 (0.67, 1.10)	1.00 (0.78, 1.30)
*p*-value for trend			0.334	0.885

The tertiles of heavy metals were classified according to blood concentrations (mg/L), with the first tertiles as the reference groups

Model 1 was adjusted for age and sex

Model 2 was further adjusted for educational achievement, monthly family income, employment status, smoking status, alcohol consumption, natural log-transformed metals, body mass index, physical activity levels, presence of diabetes, and survey year

Prevalence ratio and 95% CIs are given and visually represented by the squares and error

When stratified by physical activity levels, a significant relationship between blood Cd, Pb and hypertension was apparent among participants who were inactive and moderately active. Compared with the lowest tertile, the ORs for hypertension were increased in the 2nd and 3rd tertiles of blood Cd and Pb in both inactive and moderate active participants. These findings were not significant in participants with higher activity (Table 3).

**Table 3:** The associations between heavy metal (blood Cd and Pb) concentration and hypertension by level of physical activity

	**Cd**									
	Continuous	Q1 (< 0.64)		Q2 (0.64–1.39)		Q3 (1.39≤)		*p*-value for trend	*p*-value for interaction	
		N with/with-out hypertension	OR (95% CI)	N with/with-out hypertension	OR (95% CI)	N with/with-out hypertension	OR (95% CI)			
Inactive	1.68 (1.42, 1.99)	108/872	Reference	644/2336	1.64 (1.22, 2.21)	444/939	2.12 (1.54, 2.92)	< 0.001	0.458	
Moderate active	2.11 (1.63, 2.72)	58/458	Reference	326/1125	2.27 (1.51, 3.41)	216/491	3.40 (2.15, 5.37)	< 0.001		
Most active	1.42 (0.87, 2.34)	14/80	Reference	108/269	1.83 (0.83, 4.03)	55/136	2.21 (0.98, 4.99)	0.067		
	**Pb**									
	Continuous	Q1 (< 1.60)		Q2 (1.60–2.81)		Q3 (2.81≤)		*p*-value for trend	*p*-value for interaction	
		N with/with-out hypertension	OR (95% CI)	N with/with-out hypertension	OR (95% CI)	N with/with-out hypertension	OR (95% CI)			
Inactive	1.70 (1.34, 2.16)	154/1063	Reference	607/2222	1.25 (0.96, 1.62)	434/862	1.75 (1.29, 2.37)	< 0.001	0.114	
Moderate active	1.50 (1.00, 2.25)	45/405	Reference	293/1141	1.57 (1.02, 2.40)	262/528	2.20 (1.36, 3.58)	< 0.001		
Most active	2.24 (1.19, 4.23)	13/69	Reference	79/261	1.26 (0.54, 2.94)	85/155	2.08 (0.88, 4.92)	0.029		

Models were adjusted for age, sex (overall group only), educational achievement, monthly family income, employment status, smoking status, alcohol consumption, natural log-transformed metals, body mass index, presence of diabetes, and survey year

For further interaction analysis, individuals within the lowest tertile of Cd and Pb concentrations were defined as low exposure groups, and individuals within the 3rd tertiles were classified as a high exposure group. Compared with the most active participants with low Pb exposure, the ORs for hypertension were significantly increased in the most active participants with high Pb exposure (adjusted OR: 1.75, 95% CI: 1.07–2.86), and in inactive/ moderate active participants with high Pb exposure (adjusted OR: 1.59, 95% CI: 1.13–2.22) (Nagelkerke R^2^ = 0.261). Participants with a combination of being inactive/moderately active and high exposure to Cd had a significantly higher risk of hypertension compared with most active participants with low exposure (adjusted OR: 1.39, 95% CI: 1.01–1.92) (Nagelkerke R^2^ = 0.257). High exposure to Cd showed a positive interaction with being inactive/moderately active on hypertension. The corresponding RERI, AP and S were 0.17 (95% CI: -0.36–0.7), 0.12 (95% CI: -0.28–0.52) and 1.75 (95% CI: 1.36–2.14), respectively. About 12% of the OR of being hypertension was attributed to the interaction effect. No significant interaction of high Pb exposure with being hypertensive was found in inactive/moderately active (Table 4).

**Table 4:** Additive interactions between heavy metal (blood Cd and Pb) concentration and physical activity levels and their effect on hypertension

**Cd**					
	Low exposure		High exposure		OR (95% CI) for heavy metal exposure within strata of physical activity
	N with/with-out hypertension	OR (95% CI)	N with/with-out hypertension	OR (95% CI)	
Most active	122/349	Ref	55/136	1.27 (0.79, 2.05); *p* = 0.328	1.35 (0.81, 2.24); *p* = 0.244
Inactive/Moderate active	1135/4791	0.95 (0.70, 1.29); *p* = 0.746	660/1430	1.39 (1.01, 1.92); *p* = 0.046	1.51 (1.28, 1.76); *p* < 0.001
Measure of effect modification on additive scale: RERI (95% CI) = 0.17 (-0.36, 0.7); AP (95% CI) = 0.12 (-0.28, 0.52), S (95% CI) = 1.75 (1.36, 2.14)					
**Pb**					
	Low exposure		High exposure		OR (95% CI) for heavy metal exposure within strata of physical activity
	N with/with-out hypertension	OR (95% CI)	N with/with-out hypertension	OR (95% CI)	
Most active	92/330	Ref	85/155	1.75 (1.07, 2.86); *p* = 0.025	1.76 (1.09, 2.83); *p* = 0.02
Inactive/Moderate active	1099/4831	1.08 (0.78, 1.50); *p* = 0.628	696/1390	1.59 (1.13, 2.22); p = 0.007	1.55 (1.30, 1.86); *p* < 0.001
Measure of effect modification on additive scale: RERI (95% CI) = -0.25 (-1.09, 0.59); AP (95% CI) = -0.16 (-0.68, 0.37), S (95% CI) = 0.70 (0.23, 1.18)					

OR, odds ratio; CI, confidence interval; RERI, relative excess risk due to interaction; AP, attributable proportion due to interaction; S, synergy index

The tertiles of heavy metals were categorized as more than (High exposure) and below the 75% quartile (Low exposure)

*adjustments for age, sex (overall group only), educational achievement, monthly family income, employment status, smoking status, alcohol consumption, body mass index, presence of diabetes, and survey year

**statistically significant with RERI > 0, AP > 0, and S > 1, indicating additive interaction

The sensitivity analysis using a binomial regression confirmed no difference in the association between blood Cd and hypertension by the level of physical activity (Table S5).

## Discussion

The results of this study showed the relationship between exposure of heavy metals such as Cd and Pb and hypertension by physical activity level using a population based national data in the 2008 to 2013 KNHANES. There was a positive relationship between blood Cd and Pb and risk of hypertension. Among three level of physical activities, inactive and moderate active and blood Cd level were positively related to the risk of hypertension after adjusting age, sex, education, income, employment status, smoking, drinking, metals, BMI, diabetes and year. The same result was found in the relationship between inactive, moderate active and blood Pb level.

Our results were similar to previous studies. Heavy metals such as Cd and Pb have a relationship with hypertension by accumulating in organs such as the kidneys and destroying vascular endothelial cells [4, 14, 15]. A study based on the Korean population showed that there was a positive relationship in pre- and postmenopausal women between serum Cd, Pb, and Hg levels and the prevalence of hypertension [53]. Also, there was a positive relationship between hypertension and blood Cd with a OR of 1.51(95% confidence interval 1.13 to 2.05) comparing the highest to the lowest tertile of Cd in blood [54]. In a population based cohort in Sweden, SBP, DBP, and hypertension were positively related in the fourth quartile of blood Pb after adjustment [55]. In the Sister Study, the risk of hypertension was higher in women with exposure to Pb (PR = 1.04, 95%CI = 1.01,1.08). [56] On the other hand, opposite results exist such as the GuLF study, which showed no cross-sectional associations between blood Cd, Pb, Hg, Se levels and hypertension or blood pressure [4].

There were a positive interaction with high exposure to Cd and inactive/moderate active levels on hypertension. A study reported on Cd in sweat, the amount of Cd in sweat was higher than urine [44]. A Canadian study showed the average level of Cd in sweat was highest among blood plasma and urine [43]. Therefore, it can be speculated that the amount of sweat produced during physical activity could reduce the amount of body Cd levels and weaken the influence on hypertension. A physical activity level that leads to perfuse sweating could be recommended to people with hypertension.

PA plays an important role as a protective factor for various health problems including diabetes, metabolic diseases, and cardiovascular diseases [29–36]. PA provides positive effect on partial or total changes in vascular metabolism, muscle, and fibrinolytic systems [35]. In addition, PA has a positive effect on endothelial cell dysfunction and oxidative stress in metabolic syndrome [35]. PA increases the expression of nitric oxide synthase and EC-derived superoxide dismutase, and improves coronary EC function, vascular structure, and local and systemic fibrinolysis [37–39]. In addition, insulin sensitivity and EC function in insulin-resistant individuals can be improved by reducing body weight through PA [40]. It also reduces inflammatory cytokine levels through improvement of EC function [41], and PA make a positive impact on EC function by increasing the bioavailability of nitric oxide [42].

However, this study has limitations. We analyzed cross-sectional data, which could not investigate the casual relationship. This study applied only blood Cd and Pb levels, which indicate only the recent Cd and Pb level. In females, we did not consider whether they were taking contraceptives or not which may have an effect on amount of sweat produced. The KNHANES considered blood Cd for recent exposure [57, 58] instead of urinary or hair Cd for lifetime exposure [57, 59]. Additionally, the blood Pb levels reported here represent recent exposure and do not reflect the subject’s total body burden over the lifetime [60]. Further studies are needed to confirm the results, indicating the need for long-term cumulative exposure, and the difference between Pb and Cd in the results should be included in future studies. In addition, there is a possibility that there is a selection bias in light of the difference in general characteristics between the participants and the non-participants (Table S1). Because of the lack of available data, individual covariates such as diet factors were not included in our analyses, although these factors are likely to be associated with hypertension. Therefore, the results may be likely to be affected by residual bias.

## Conclusion

Cd and Pb were positively related to hypertension. In the subgroup analysis by physical activity, blood Cd and Pb were positively related with the risk of hypertension in inactive and moderate active levels. However, interaction existed only with Cd and physical activity.

## Electronic supplementary material

Below is the link to the electronic supplementary material.

Supplementary Material 1

## Data Availability

The datasets analyzed during the current study are publicly available for free on the KNHANES website (https://knhanes.kdca.go.kr/knhanes/sub03/sub03_02_05.do).

## List of abbreviations

CRF: cardiorespiratory fitness
KNHANES: The Korea National Health and Nutrition Examination Survey
BMI: body mass index
IPAQ: International Physical Activity Questionnaire
SBP: systolic blood pressure
DBP: diastolic blood pressure
RERI: risk due to interaction
AP: attributable proportion due to interaction
S: synergy index
