# Supplementary material for: Interaction between blood cadmium and lead concentration and physical activity on hypertension from the Korean national health and nutrition examination survey in 2008–2013
Source: BMC Public Health. 2023 Apr 17;23:703. doi: 10.1186/s12889-023-15614-x (PMC10111815; doi:10.1186/s12889-023-15614-x)
Supplement: Supplementary file 1 — Supplementary Material 1 [file 12889_2023_15614_MOESM1_ESM.docx]

**STable 1.** Comparison of characteristics between participants and non-participants

| Variables | N (%), Mean±SE | | |  | |
| --- | --- | --- | --- | --- | --- |
|  | Participants  (N=8,678) | Non-participants  (N=4,835) | *p-*value^*^ | |  |
| **Age** (year) | 43.59 ± 0.20 | 39.89 ± 0.37 | <0.001 | |  |
| <20 | - | 1472 (25.64) |  | |  |
| 20-39 | 3710 (42.99) | 1118 (24.60) |  | |  |
| 40-59 | 3538 (40.63) | 1273 (27.80) |  | |  |
| 60+ | 1430 (16.38) | 972 (21.95) |  | |  |
| **Gender** |  |  |  | |  |
| Male | 4359 (50.34) | 2360 (48.83) | 0.139 | |  |
| Female | 4319 (49.66) | 2475 (51.17) |  | |  |
| **Educational achievement** |  |  |  | |  |
| High school or less | 5503 (65.49) | 3839 (84.32) | <0.001 | |  |
| College or more | 3160 (34.51) | 736 (15.69) |  | |  |
| **Monthly family income** |  |  |  | |  |
| High | 2731 (30.48) | 1204 (23.76) | <0.001 | |  |
| Middle | 4741 (55.45) | 2644 (55.22) |  | |  |
| Low | 1113 (13.94) | 912 (21.02) |  | |  |
| **Alcohol consumption** |  |  |  | |  |
| No | 1818 (21.69) | 2070 (42.04) | <0.001 | |  |
| Yes | 6843 (78.31) | 2499 (57.96) |  | |  |
| **Smoking status** |  |  |  | |  |
| Never smoker | 4749 (54.85) | 1697 (54.79) | 0.995 | |  |
| Moderate smoker (< 5 packs) | 294 (3.29) | 101 (3.26) |  | |  |
| Heavy smoker (≥5 packs) | 3632 (41.86) | 1312 (41.95) |  | |  |
| **Body mass index** (kg/m^2^) | 23.75 ± 0.05 | 22.95 ± 0.07 | <0.001 | |  |
| **Physical activity levels** |  |  |  | |  |
| Inactive | 5342 (61.35) | 540 (57.34) | 0.002 | |  |
| Moderate active | 2674 (31.09) | 358 (37.16) |  | |  |
| Most active | 662 (7.56) | 56 (5.50) |  | |  |
| **Presence of Hypertension** |  |  |  | |  |
| No | 6706 (77.28) | 3794 (77.29) | 0.591 | |  |
| Yes | 1972 (22.72) | 1019 (22.71) |  | |  |
| **Blood Cd (μg/L)**, GM^a^ (95% CI) | 0.92 (0.91, 0.94) | 0.75 (0.73, 0.77) | <0.001 | |  |
| **Blood Pb (μg/dL)**, GM^a^ (95% CI) | 2.11 (2.09, 2.14) | 1.91 (1.88, 1.95) | <0.001 | |  |

^*^p-value for comparison between non- participants and participants estimates based on t-tests for continuous variables and chi-square tests for categorical variables.

Data are presented as weighted mean±standard error or frequency (weighted percentage)

^a^ GM, geometric mean; CI, confidence interval; SE, standard error.

**STable 2.** General characteristics of study participants

| Variables | N (%), Mean±SE | | |
| --- | --- | --- | --- |
|  | Overall | Non-Hypertension | Hypertension |
|  | 8,678 | 6,706 (77.28%) | 1,972 (22.72%) |
| **Blood Cd (μg/L)** |  |  |  |
| Geometric Mean (95% CI) | 0.92 (0.91, 0.94) | 0.87 (0.85, 0.88) | 1.13 (1.10, 1.17) |
| **Blood Pb (μg/dL)** |  |  |  |
| Geometric Mean (95% CI) | 2.11 (2.09, 2.14) | 2.03 (2.00, 2.05) | 2.44 (2.38, 2.50) |
| **Age** |  |  |  |
| Age (year), Mean±SE | 43.59 ± 0.20 | 40.23 ± 0.19 | 54.70 ± 0.44 |
| 19-39 | 3624 (42.23) | 3365 (50.94) | 259 (13.80) |
| 40-59 | 3538 (41.17) | 2593 (39.40) | 945 (46.95) |
| 60+ | 1430 (16.60) | 664 (9.66) | 766 (39.25) |
| **Gender** |  |  |  |
| Male | 4359 (50.34) | 3185 (48.44) | 1174 (56.62) |
| Female | 4319 (49.66) | 3521 (51.56) | 798 (43.38) |
| **Educational achievement** |  |  |  |
| High school or less | 5503 (65.49) | 3967 (61.07) | 1536 (80.09) |
| College or more | 3160 (34.51) | 2727 (38.93) | 433 (19.91) |
| **Monthly family income** |  |  |  |
| High | 2731 (30.48) | 2236 (32.72) | 495 (23.56) |
| Middle | 4741 (55.45) | 3724 (56.31) | 1017 (52.58) |
| Low | 1113 (13.94) | 689 (10.97) | 424 (23.85) |
| **Employment status** |  |  |  |
| Unemployed | 3074 (35.60) | 2343 (34.60) | 731 (38.90) |
| Employed | 5564 (64.40) | 4331 (65.40) | 1233 (61.10) |
| **Smoking status** |  |  |  |
| Never smoker | 4749 (54.85) | 3813 (56.32) | 936 (50) |
| Moderate smoker (< 5 packs) | 294 (3.29) | 256 (3.8) | 38 (1.6) |
| Heavy smoker (≥5 packs) | 3632 (41.86) | 2635 (39.88) | 997 (48.41) |
| **Body mass index** |  |  |  |
| Body mass index, Mean±SE | 23.75 ± 0.05 | 23.31 ± 0.05 | 25.21 ± 0.10 |
| Acceptable weight (<23 kg/m^2^) | 4848 (56.20) | 3330 (49.27) | 500 (25.72) |
| Overweight/Obese (≥23 kg/m^2^) | 3830 (43.80) | 3376 (50.73) | 1472 (74.28) |
| **Physical activity levels** |  |  |  |
| Inactive | 5342 (61.35) | 4147 (61.31) | 1195 (61.49) |
| Moderate active | 2674 (31.09) | 2074 (31.41) | 600 (30.01) |
| Most active | 662 (7.56) | 485 (7.27) | 177 (8.49) |
| **Alcohol consumption** |  |  |  |
| No | 1818 (21.69) | 1316 (19.72) | 502 (28.21) |
| Yes | 6843 (78.31) | 5377 (80.28) | 1466 (71.79) |
| **Presence of diabetes** |  |  |  |
| No | 8037 (92.70) | 6415 (95.65) | 1622 (82.95) |
| Yes | 641 (7.30) | 291 (4.35) | 350 (17.05) |
| **Survey year** |  |  |  |
| 2008 | 1400 (15.74) | 1093 (16.13) | 307 (14.43) |
| 2009 | 1469 (16.79) | 1058 (15.90) | 411 (19.75) |
| 2010 | 1454 (16.84) | 1104 (16.59) | 350 (17.66) |
| 2011 | 1469 (16.83) | 1102 (16.17) | 367 (19.02) |
| 2012 | 1420 (16.15) | 1067 (15.39) | 353 (18.68) |
| 2013 | 1466 (17.65) | 1282 (19.83) | 184 (10.47) |

Data are presented as weighted mean±standard error or frequency (weighted percentage)

GM, geometric mean; CI, confidence interval; SE, standard error.

**STable 3.** Age-adjusted geometric mean of blood Cd by participants’ characteristics

| Variables | Blood Cd (μg/L) | | | | | |
| --- | --- | --- | --- | --- | --- | --- |
|  | Overall | | Non-Hypertension | | Hypertension | |
|  | GM^a^ (95% CI) | *p-*value | GM^a^ (95% CI) | *p*-value | GM^a^ (95% CI) | *p*-value |
| **Age** |  |  |  |  |  |  |
| 19-39 | 0.70 (0.68, 0.72) | <0.001 | 0.69 (0.67, 0.71) | <0.001 | 0.82 (0.74, 0.90) | <0.001 |
| 40-59 | 1.11 (1.09, 1.13) |  | 1.08 (1.06, 1.11) |  | 1.19 (1.14, 1.23) |  |
| 60+ | 1.20 (1.16, 1.24) |  | 1.19 (1.15, 1.24) |  | 1.20 (1.15, 1.26) |  |
| **Gender** |  |  |  |  |  |  |
| Male | 0.95 (0.92, 0.98) | <0.001 | 0.92 (0.89, 0.95) | <0.001 | 1.01 (0.96, 1.06) | <0.001 |
| Female | 1.12 (1.09, 1.15) |  | 1.08 (1.05, 1.12) |  | 1.33 (1.27, 1.39) |  |
| **Educational achievement** |  |  |  |  |  |  |
| High school or less | 1.09 (1.07, 1.12) | <0.001 | 1.05 (1.01, 1.08) | <0.001 | 1.22 (1.17, 1.26) | <0.001 |
| College or more | 0.87 (0.84, 0.90) |  | 0.87 (0.83, 0.90) |  | 0.90 (0.84, 0.95) |  |
| **Monthly family income** |  |  |  |  |  |  |
| High | 1.18 (1.13, 1.23) | <0.001 | 0.92 (0.88, 0.96) | <0.001 | 1.06 (1.00, 1.13) | <0.001 |
| Middle | 1.01 (0.98, 1.03) |  | 0.98 (0.95, 1.02) |  | 1.11 (1.06, 1.16) |  |
| Low | 0.94 (0.91, 0.98) |  | 1.11 (1.05, 1.18) |  | 1.33 (1.25, 1.41) |  |
| **Employment status** |  |  |  |  |  |  |
| Unemployed | 1.03 (1.00, 1.06) | 0.271 | 0.98 (0.94, 1.02) | 0.022 | 1.23 (1.17, 1.28) | 0.001 |
| Employed | 1.05 (1.02, 1.08) |  | 1.02 (0.99, 1.06) |  | 1.10 (1.05, 1.16) |  |
| **Smoking status** |  |  |  |  |  |  |
| Never smoker | 0.99 (0.96, 1.02) | <0.001 | 0.95 (0.92, 0.98) | <0.001 | 1.17 (1.11, 1.22) | 0.003 |
| Moderate smoker (< 5 packs) | 0.80 (0.74, 0.86) |  | 0.79 (0.72, 0.86) |  | 0.90 (0.77, 1.04) |  |
| Heavy smoker (≥5 packs) | 1.13 (1.10, 1.16) |  | 1.10 (1.06, 1.14) |  | 1.16 (1.11, 1.21) |  |
| **Body mass index** |  |  |  |  |  |  |
| Acceptable weight (<23 kg/m^2^) | 0.99 (0.97, 1.02) | <0.001 | 0.97 (0.93, 1.01) | 0.004 | 1.21 (1.14, 1.27) | 0.124 |
| Overweight/Obese (≥23 kg/m^2^) | 1.07 (1.04, 1.10) |  | 1.03 (0.99, 1.06) |  | 1.14 (1.10, 1.19) |  |
| **Physical activity levels** |  |  |  |  |  |  |
| Inactive | 1.04 (1.01, 1.07) | 0.088 | 1.00 (0.97, 1.04) | 0.039 | 1.17 (1.12, 1.21) | 0.852 |
| Moderate active | 1.02 (0.98, 1.05) |  | 0.98 (0.94, 1.02) |  | 1.15 (1.09, 1.23) |  |
| Most active | 1.09 (1.03, 1.16) |  | 1.07 (1.00, 1.15) |  | 1.13 (0.99, 1.28) |  |
| **Alcohol consumption** |  |  |  |  |  |  |
| No | 1.11 (1.07, 1.14) | <0.001 | 1.07 (1.03, 1.12) | <0.001 | 1.22 (1.16, 1.29) | 0.021 |
| Yes | 1.00 (0.98, 1.03) |  | 0.97 (0.94, 1.00) |  | 1.13 (1.08, 1.18) |  |
| **Presence of diabetes** |  |  |  |  |  |  |
| No | 1.02 (1.00, 1.05) | <0.001 | 0.99 (0.96, 1.02) | 0.003 | 1.16 (1.12, 1.21) | 0.768 |
| Yes | 1.14 (1.09, 1.20) |  | 1.11 (1.03, 1.20) |  | 1.15 (1.08, 1.22) |  |
| **Survey year** |  |  |  |  |  |  |
| 2008 | 1.01 (0.96, 1.06) | <0.001 | 0.98 (0.93, 1.04) | <0.001 | 1.10 (1.02, 1.18) | 0.094 |
| 2009 | 1.02 (0.97, 1.07) |  | 0.97 (0.91, 1.03) |  | 1.13 (1.05, 1.21) |  |
| 2010 | 1.13 (1.09, 1.17) |  | 1.01 (1.05, 1.15) |  | 1.23 (1.16, 1.31) |  |
| 2011 | 1.09 (1.05, 1.14) |  | 1.06 (1.01, 1.12) |  | 1.19 (1.11, 1.28) |  |
| 2012 | 1.05 (1.00, 1.11) |  | 1.01 (0.96, 1.07) |  | 1.17 (1.08, 1.28) |  |
| 2013 | 0.92 (0.88, 0.96) |  | 0.90 (0.86, 0.95) |  | 1.11 (1.01, 1.21) |  |

^a^ GM, geometric mean; CI, confidence interval; SE, standard error. GMs were adjusted for age group.

**STable 4.** Age-adjusted geometric mean of blood Pb by participants’ characteristics

| Variables | Blood Pb (μg/dL) | | | | | |
| --- | --- | --- | --- | --- | --- | --- |
|  | Overall | | Non-Hypertension | | Hypertension | |
|  | GM^a^ (95% CI) | *p*-value | GM^a^ (95% CI) | *p*-value | GM^a^ (95% CI) | *p*-value |
| **Age** |  |  |  |  |  |  |
| 19-39 | 1.83 (1.80, 1.86) | <0.001 | 1.80 (1.77, 1.83) | <0.001 | 2.21 (2.10, 2.32) | <0.001 |
| 40-59 | 2.37 (2.33, 2.40) |  | 2.28 (2.24, 2.32) |  | 2.62 (2.54, 2.70) |  |
| 60+ | 2.33 (2.26, 2.40) |  | 2.35 (2.25, 2.46) |  | 2.31 (2.23, 2.41) |  |
| **Gender** |  |  |  |  |  |  |
| Male | 2.62 (2.56, 2.67) | <0.001 | 2.56 (2.48, 2.64) | <0.001 | 2.74 (2.65, 2.83) | <0.001 |
| Female | 1.90 (1.86, 1.94) |  | 1.87 (1.81, 1.93) |  | 2.08 (2.01, 2.16) |  |
| **Educational achievement** |  |  |  |  |  |  |
| High school or less | 2.25 (2.20, 2.30) | <0.001 | 2.23 (2.15, 2.31) | <0.001 | 2.39 (2.32, 2.46) | 0.819 |
| College or more | 2.02 (1.96, 2.08) |  | 2.01 (1.93, 2.09) |  | 2.37 (2.25, 2.50) |  |
| **Monthly family income** |  |  |  |  |  |  |
| High | 2.26 (2.19, 2.34) | 0.026 | 2.25 (2.15, 2.37) | 0.040 | 2.37 (2.26, 2.48) | 0.874 |
| Middle | 2.17 (2.12, 2.23) |  | 2.15 (2.08, 2.23) |  | 2.37 (2.29, 2.47) |  |
| Low | 2.14 (2.08, 2.21) |  | 2.12 (2.04, 2.21) |  | 2.40 (2.27, 2.54) |  |
| **Employment status** |  |  |  |  |  |  |
| Unemployed | 2.40 (2.34, 2.45) | <0.001 | 2.37 (2.29, 2.45) | <0.001 | 2.59 (2.49, 2.69) | <0.001 |
| Employed | 2.01 (1.96, 2.06) |  | 2.00 (1.93, 2.07) |  | 2.18 (2.10, 2.26) |  |
| **Smoking status** |  |  |  |  |  |  |
| Never smoker | 1.94 (1.90, 1.99) | <0.001 | 1.92 (1.86, 1.98) | <0.001 | 2.13 (2.05, 2.20) | <0.001 |
| Moderate smoker (< 5 packs) | 2.12 (2.01, 2.24) |  | 2.09 (1.96, 2.22) |  | 2.57 (2.25, 2.94) |  |
| Heavy smoker (≥5 packs) | 2.66 (2.60, 2.72) |  | 2.62 (2.54, 2.71) |  | 2.76 (2.67, 2.85) |  |
| **Body mass index** |  |  |  |  |  |  |
| Acceptable weight (<23 kg/m^2^) | 2.02 (1.97, 2.08) | <0.001 | 2.03 (1.96, 2.11) | <0.001 | 2.31 (2.20, 2.42) | 0.087 |
| Overweight/Obese (≥23 kg/m^2^) | 2.32 (2.27, 2.37) |  | 2.31 (2.23, 2.39) |  | 2.41 (2.34, 2.49) |  |
| **Physical activity levels** |  |  |  |  |  |  |
| Inactive | 2.12 (2.08, 2.17) | <0.001 | 2.10 (2.03, 2.17) | <0.001 | 2.32 (2.24, 2.40) | <0.001 |
| Moderate active | 2.31 (2.25, 2.37) |  | 2.29 (2.20, 2.39) |  | 2.45 (2.46, 2.55) |  |
| Most active | 2.49 (2.39, 2.60) |  | 2.44 (2.31, 2.57) |  | 2.70 (2.50, 2.92) |  |
| **Alcohol consumption** |  |  |  |  |  |  |
| No | 2.02 (1.97, 2.08) | <0.001 | 2.04 (1.96, 2.12) | <0.001 | 2.07 (2.98, 2.17) | <0.001 |
| Yes | 2.29 (2.24, 2.34) |  | 2.24 (2.16, 2.31) |  | 2.58 (2.50, 2.66) |  |
| **Presence of diabetes** |  |  |  |  |  |  |
| No | 2.18 (2.13, 2.23) | 0.010 | 2.16 (2.09, 2.23) | 0.018 | 2.40 (2.33, 2.48) | 0.186 |
| Yes | 2.33 (2.21, 2.45) |  | 2.35 (2.17, 2.54) |  | 2.29 (2.15, 2.45) |  |
| **Survey year** |  |  |  |  |  |  |
| 2008 | 2.40 (2.32, 2.48) | <0.001 | 2.39 (2.28, 2.50) | <0.001 | 2.57 (2.44, 2.70) | <0.001 |
| 2009 | 2.37 (2.29, 2.46) |  | 2.31 (2.21, 2.41) |  | 2.59 (2.46, 2.73) |  |
| 2010 | 2.26 (2.19, 2.33) |  | 2.24 (2.15, 2.34) |  | 2.41 (2.30, 2.53) |  |
| 2011 | 2.17 (2.09, 2.25) |  | 2.15 (2.05, 2.25) |  | 2.30 (2.15, 2.47) |  |
| 2012 | 2.05 (1.98, 2.13) |  | 2.01 (1.91, 2.12) |  | 2.25 (2.13, 2.38) |  |
| 2013 | 1.96 (1.90, 2.03) |  | 1.97 (1.89, 2.06) |  | 2.18 (2.05, 2.32) |  |

^a^ GM, geometric mean; CI, confidence interval; SE, standard error. GMs were adjusted for age group

**STable 5.** Additive interactions between heavy metal (blood Cd and Pb) concentration and physical activity levels and their effect on hypertension

| **Cd** | | | | | |
| --- | --- | --- | --- | --- | --- |
|  | Low exposure | | High exposure | | OR (95% CI) for heavy metal exposure within strata of physical activity |
|  | N with/with-out hypertension | OR (95% CI) | N with/with-out hypertension | OR (95% CI) |  |
| Most active | 122/349 | Ref | 55/136 | 1.27 (0.79, 2.05); *p*=0.326 | 1.35 (0.82, 2.23); *p*=0.24 |
| Inactive/Moderate active | 1135/4791 | 0.95 (0.70, 1.29); *p*=0.746 | 660/1430 | 1.39 (1.00, 1.92); *p*=0.047 | 1.50 (1.28, 1.76); *p*<0.001 |
| Measure of effect modification on additive scale: RERI (95% CI) = 0.17 (-0.36, 0.7); AP (95% CI) = 0.12 (-0.28, 0.52), S (95% CI) = 1.75 (1.36, 2.14) | | | | | |
|  | | | | | |
| **Pb** | | | | | |
|  | Low exposure | | High exposure | | OR (95% CI) for heavy metal exposure within strata of physical activity |
|  | N with/with-out hypertension | OR (95% CI) | N with/with-out hypertension | OR (95% CI) |  |
| Most active | 92/330 | Ref | 85/155 | 1.75 (1.08, 2.85); *p*=0.024 | 1.76 (1.09, 2.83); *p*=0.02 |
| Inactive/Moderate active | 1099/4831 | 1.08 (0.78, 1.50); *p*=0.628 | 696/1390 | 1.59 (1.13, 2.22); *p*=0.007 | 1.55 (1.30, 1.86); *p*<0.001 |
| Measure of effect modification on additive scale: RERI (95% CI) = -0.25 (-1.08, 0.58); AP (95% CI) = -0.16 (-0.67, 0.36), S (95% CI) = 0.70 (0.23, 1.17) | | | | | |

OR odds ratio; CI, confidence interval; RERI, relative excess risk due to interaction; AP, attributable proportion due to interaction; S, synergy index

The tertiles of heavy metals were categorized as more than (High exposure) and below the 75% quartile (Low exposure).

*adjustments for age, sex (overall group only), educational achievement, monthly family income, employment status, smoking status, alcohol consumption, body mass index, presence of diabetes, and survey year.

**statistically significant with RERI > 0, AP > 0, and S > 1, indicating additive interaction

***The binomial regression models were used to evaluate the association between heavy metal concentration, physical activity levels and hypertension.
